# Antimicrobial resistance: from global agenda to national strategic plan, Thailand

**DOI:** 10.2471/BLT.16.179648

**Published:** 2017-05-11

**Authors:** Viroj Tangcharoensathien, Wanchai Sattayawutthipong, Sukhum Kanjanapimai, Wantanee Kanpravidth, Richard Brown, Angkana Sommanustweechai

**Affiliations:** a International Health Policy Program, Ministry of Public Health, Soi Satharanasook 6, Tiwanon Road, Nonthaburi 11000, Thailand.; b Food and Drug Administration, Ministry of Public Health, Nonthaburi, Thailand.; c Department of Medical Science, Ministry of Public Health, Nonthaburi, Thailand.; d Food and Agriculture Organization Regional Office for Asia and the Pacific, Bangkok, Thailand.; e World Health Organization Country Office, Ministry of Public Health, Nonthaburi, Thailand.

## Abstract

**Problem:**

In Thailand, antimicrobial resistance has formed a small component of national drug policies and strategies on emerging infectious diseases. However, poor coordination and a lack of national goals and monitoring and evaluation platforms have reduced the effectiveness of the corresponding national actions.

**Approach:**

On the basis of local evidence and with the strong participation of relevant stakeholders, the first national strategic plan on antimicrobial resistance has been developed in Thailand.

**Local setting:**

Before the development of the plan, ineffective coordination meant that antimicrobial resistance profiles produced at sentinel hospitals were not used effectively for clinical decision-making. There was no integrated system for the surveillance of antimicrobial resistance, no system for monitoring consumption of antimicrobial drugs by humans, livestock and pets and little public awareness of antimicrobial resistance.

**Relevant changes:**

In August 2016, the Thai government endorsed a national strategic plan on antimicrobial resistance that comprised six strategic actions and five targets. A national steering committee guides the plan’s implementation and a module to assess the prevalence of household antibiotic use and antimicrobial resistance awareness has been embedded into the biennial national health survey. A national system for the surveillance of antimicrobial consumption has also been initiated.

**Lessons learnt:**

Strong political commitment, national ownership and adequate multisectoral institutional capacities will be essential for the effective implementation of the national plan. A robust monitoring and evaluation platform now contributes to evidence-based interventions. An integrated system for the surveillance of antimicrobial resistance still needs to be established.

## Résumé

**Problème:**

En Thaïlande, le problème de résistance aux antimicrobiens était partiellement pris en compte dans les stratégies et politiques nationales liées à la consommation de médicaments et destinées à lutter contre les maladies infectieuses émergentes. Mais des problèmes de coordination et l'absence d'objectifs nationaux et de plateformes de surveillance et d'évaluation limitaient l'efficacité de ces initiatives nationales.

**Approche:**

À partir des données factuelles disponibles sur la situation locale et grâce à une importante participation des parties prenantes concernées, la Thaïlande a élaboré son premier plan stratégique national sur la résistance aux antimicrobiens.

**Environnement local:**

Avant la conception de ce plan, une coordination inefficace faisait que les profils de résistance aux antimicrobiens produits dans les hôpitaux sentinelles n'étaient pas efficacement utilisés dans la prise de décisions cliniques. Il n'existait aucun système intégré de surveillance de la résistance aux antimicrobiens, aucun système de suivi de la consommation d’antimicrobiens par les humains, le bétail et les animaux domestiques, et la population était peu sensibilisée au problème de résistance aux antimicrobiens.

**Changements significatifs:**

En août 2016, le gouvernement thaïlandais a validé un plan stratégique national sur la résistance aux antimicrobiens, comprenant six actions stratégiques et cinq objectifs. Un comité de pilotage national a été constitué pour guider la mise en œuvre du plan, et un module destiné à évaluer la prévalence de l'utilisation des antibiotiques dans les ménages et la sensibilisation à la résistance aux antimicrobiens a été intégré dans l'enquête nationale sur la santé réalisée tous les deux ans. Un système national pour la surveillance de la consommation d'antimicrobiens a également été lancé.

**Leçons tirées:**

Un engagement politique fort, une mobilisation nationale et des capacités institutionnelles multisectorielles appropriées seront essentiels pour une mise en œuvre efficace du plan national. Une solide plateforme d'évaluation et de surveillance contribue désormais à fonder les interventions sur des données factuelles. Mais un système intégré pour la surveillance de la résistance aux antimicrobiens doit encore être créé.

## Resumen

**Situación:**

En Tailandia, la resistencia a los antimicrobianos ha formado un pequeño componente de las políticas y estrategias de medicamentos nacionales sobre las enfermedades infecciosas emergentes. No obstante, la escasa coordinación y la ausencia de objetivos nacionales y plataformas de control y evaluación han reducido la efectividad de las medidas nacionales correspondientes.

**Enfoque:**

Sobre la base de las pruebas locales y con la gran participación de partes interesadas relevantes, se ha desarrollado en Tailandia el primer plan estratégico nacional sobre la resistencia a los antimicrobianos.

**Marco regional:**

Antes de desarrollar el plan, la mala coordinación provocó que los perfiles de resistencia a los antimicrobianos producidos en los hospitales centinela no se utilizasen de forma adecuada para la toma de decisiones clínicas. No existía un sistema integrado para la vigilancia de la resistencia a los antimicrobianos, ni un sistema para el control del consumo de medicamentos antimicrobianos por parte de humanos, ganado y mascotas, ni apenas concienciación pública sobre la resistencia a los antimicrobianos.

**Cambios importantes:**

En agosto de 2016, el gobierno tailandés aprobó un plan estratégico nacional sobre la resistencia a los antimicrobianos que estaba formado por seis medidas estratégicas y cinco objetivos. Un comité directivo nacional dirige la implementación del plan y un módulo para evaluar la prevalencia del uso doméstico de antibióticos y se ha integrado la concienciación sobre la resistencia a los antimicrobianos en la encuesta nacional bienal sobre salud. También se ha iniciado un sistema nacional para la vigilancia del consumo de antimicrobianos.

**Lecciones aprendidas:**

Un fuerte compromiso político, una propiedad nacional y las capacidades institucionales multisectoriales adecuadas serán esenciales para la implementación eficaz del plan nacional. Ahora, una sólida plataforma de control y evaluación contribuye a las intervenciones basadas en pruebas. Sin embargo, aún debe establecerse un sistema integrado para la vigilancia de la resistencia a los antimicrobianos.

## ملخص

المشكلة شكلت مقاومة الميكروبات في تايلند عنصرًا ضئيلاً من سياسات واستراتيجيات الأدوية الوطنية بشأن الأمراض المعدية الناشئة. ومع ذلك، فقد أدى ضعف التنسيق وغياب الأهداف الوطنية ومنصات الرصد والتقييم إلى تقليص فعالية الإجراءات الوطنية المقابلة.

الأسلوب استنادًا إلى الأدلة المحلية وبمشاركة قوية من أصحاب المصلحة المعنيين، فقد تم وضع أول خطة استراتيجية وطنية بشأن مقاومة الميكروبات في تايلند.

المواقع ****المحلية قبل وضع الخطة، كان التنسيق غير الفعال يعني أن أنماط مقاومة الميكروبات الناتجة في المستشفيات الخفرية لم تكن تُستخدم بشكل فعال لاتخاذ القرار السريري. ولم يكن هناك نظام متكامل لمراقبة مقاومة الميكروبات، ولا نظام لرصد استهلاك العقاقير المقاومة للميكروبات من قبل البشر والماشية والحيوانات الأليفة، بالإضافة إلى ضعف الوعي العام بمقاومة الميكروبات.

التغيّرات ****ذات ****الصلة أقرت الحكومة التايلندية خطة استراتيجية وطنية بشأن مقاومة الميكروبات والتي تضم ستة إجراءات استراتيجية وخمسة أهداف وذلك في شهر آب/أغسطس من عام 2016. وتوجد لجنة توجيهية وطنية معنية بتنفيذ الخطة، وقد تم تضمين وحدة لتقييم مدى انتشار استخدام المضادات الحيوية بين العائلات والوعي بمقاومة الميكروبات في المسح الصحي الوطني الذي يُجرى كل سنتين. كما بدأ العمل بنظام وطني لمراقبة استهلاك مقاومة الميكروبات.

الدروس ****المستفادة سيكون الالتزام السياسي القوي والمسؤولية الوطنية والقدرات المؤسسية الكافية متعددة القطاعات أمرًا أساسيًا للتنفيذ الفعال للخطة الوطنية. وتساهم الآن منصة قوية للرصد والتقييم في التدخلات القائمة على الأدلة. ولا يزال يتعين إنشاء نظام متكامل لمراقبة مقاومة الميكروبات.

## 摘要

**问题:**

在泰国,抗菌素耐药性已成为针对新发现传染疾病的国家药物政策和策略的一小部分。 然而,协调不力、缺乏国家目标以及监测和评估平台降低了相应国家行动的有效性。

**方法:**

基于当地实情和在相关利益攸关方的大力参与下,首个国家抗菌素耐药性战略计划已于泰国制定。

**当地状况:**

该计划制定之前,无效协调指在哨点医院生成的抗菌素耐药性资料未被有效地用于临床决策。 没有用于监测抗菌素耐药性的综合系统,没有监测人类、家畜和宠物抗菌药物消耗的系统以及公众缺少对抗菌素耐药性的认识。

**相关变化:**

2016 年 8 月,泰国政府批准了一项抗菌素耐药性国家战略计划,其中包括六项战略行动和五个目标。 国家指导委员会指导该计划的实施,并将一个评估家庭的抗生素使用情况及对抗菌素抗药性的认识的模块纳入为期两年的国家健康调查。 还建立了一个国家抗菌药物消耗监测系统。

**经验教训:**

强有力的政治承诺、国家所有权和充分的多部门机构职能对于有效执行该项国家计划至关重要。 如今的监测和评估平台有助于制定循证干预措施。 仍需建立一个抗菌素耐药性综合监测系统。

## Резюме

**Проблема:**

В Таиланде проблема устойчивости возбудителей к противомикробным препаратам стала одним из второстепенных направлений национальной политики и стратегии в отношении применения лекарственных препаратов при вновь возникающих инфекционных заболеваниях. Однако слабая координация и отсутствие национальных целей и платформ мониторинга и оценки снизили эффективность соответствующих национальных мероприятий.

**Подход:**

С учетом местных данных и при активном участии соответствующих заинтересованных сторон в Таиланде был разработан первый национальный стратегический план по решению проблемы с устойчивостью возбудителей к противомикробным препаратам.

**Местные условия:**

До разработки плана неэффективная координация означала, что профили устойчивости возбудителей к противомикробным препаратам, составленные в дозорных больницах, неэффективно использовались при принятии клинических решений. Отсутствовала единая система для наблюдения за устойчивостью возбудителей к противомикробным препаратам, а также система мониторинга применения противомикробных препаратов у людей, сельскохозяйственных и домашних животных, и информированность общественности об устойчивости возбудителей к противомикробным препаратам была недостаточной.

**Осуществленные перемены:**

В августе 2016 года Правительство Таиланда одобрило национальный стратегический план по решению проблемы устойчивости возбудителей к противомикробным препаратам, который включал шесть стратегических мероприятий и пять целей. Национальный управляющий комитет руководит реализацией плана, и в двухгодичный обзор общественного здравоохранения включен встроенный модуль оценки распространенности использования антибиотиков в домашних условиях и осведомленности общественности о микробной устойчивости. Также была введена национальная система наблюдения за применением антимикробных препаратов.

**Выводы:**

Для эффективной реализации национального плана крайне важны решительная политическая приверженность, национальная ответственность и наличие соответствующего многосекторального институционального потенциала. Надежная платформа мониторинга и оценки в настоящее время вносит свой вклад в научно обоснованные вмешательства. Комплексную систему надзора за устойчивостью возбудителей к противомикробным препаратам все еще предстоит создать.****

## Introduction

Antimicrobial resistance poses a serious security threat to global health. In Thailand alone, such resistance is estimated to have caused 38 000 deaths and an economic loss of 1.2 billion United States dollars (US$) in 2010.^1^ Subsequent global attention and national concern pushed the Thai government to take action against antimicrobial resistance.

The increased prevalence and global spread of drug-resistant microorganisms are alarming.^2^ Antimicrobial resistance is recognized as a key security threat to global health. By 2050 – if effective interventions against antimicrobial resistance are not made – 10 million deaths and an economic loss of US$ 100 trillion may occur annually as the result of such resistance.^3^ In 2015, the Sixty-eighth World Health Assembly adopted a *Global action plan for antimicrobial resistance* and called on each Member State to develop and implement a corresponding context-specific national plan.^4^ Discussion of antimicrobial resistance at the United Nations General Assembly in September 2016 led to a political declaration that endorsed the implementation of the global action plan using a One Health approach.^5^^,^^6^

Thailand has previously addressed antimicrobial resistance with fragmented approaches. For example, antimicrobial resistance formed a small component of the *National drug development strategy (2012–2016) *and the *National strategic plan on emerging infectious disease (2013–2016)*. Although several relevant working groups were established, no platform for coordination existed and only very limited policy guidance. None of the working groups established national indicators, systems for monitoring and evaluation or targets. Fragmentation and the lack of both coordination and concerted efforts hampered progress.

## Local setting

In Thailand, although the widespread availability of antibiotics from private pharmacies leads to frequent self-medication in households, no systems exist either to monitor the human consumption of antibiotics or to assess the general public’s knowledge and perceptions of – or attitudes towards –antibiotics and antimicrobial resistance. A national system for the surveillance of antimicrobial resistance in humans was initiated by the ministry of public health’s department of medical sciences in 1998^7^ but this system covered only 92 (9%) of the country’s 1027 hospitals by 2017. Furthermore, the use of the antimicrobial resistance profiles and prevalence determined at these 92 sentinel hospitals to guide clinical decision-making by pharmacists or physicians appears to be uncommon.

Thailand’s department of livestock development and food and drug administration have conducted some small-scale monitoring of antimicrobial resistance in livestock, and some universities have undertaken some research on this topic. However, nobody has conducted a systematic evaluation of the emergence or prevalence of antimicrobial resistance in livestock.

The surveillance of antimicrobial resistance in – and consumption of antimicrobial drugs by – humans, livestock and pets is key to assessing consumption trends, evaluating the outcomes of interventions and creating national benchmarks for use in international comparisons. However, despite the high estimates of mean global antibiotic consumption in humans – e.g. more than 20 standard units per capita^8^ – and in chickens and pigs – e.g. more than 250 kg per 10 km^2^ of land used to rear such animals^9^ – there are, as yet, no national policy decisions to support the development of a comprehensive surveillance system in Thailand.

## Approach

In Thailand, the development of a *National strategic plan on antimicrobial resistance* at the end of 2015 was perhaps the most important step towards addressing the fragmentation in the investigation of antimicrobial resistance and the lack of associated systematic interventions. The many stakeholders involved in this development included those working in the agriculture, animal health and human health sectors, civil society organizations, the Food and Agricultural Organization of the United Nations (FAO) and the World Health Organization (WHO). The implementation of the plan was facilitated by the creation of a national coordinating committee for antimicrobial resistance, which had the support of a cross-ministerial joint secretariat. The plan was based on both the relevant local evidence that was available – e.g. on antimicrobial resistance in hospitals, livestock and food products and on resistance-attributable mortality and economic losses – and on the relevant information available from other countries – e.g. on policies and practices that curb antimicrobial consumption and systems for the surveillance of antimicrobial resistance.

In December 2015, in response to the need for evidence-based policy formulation and multisectoral actions, antimicrobial resistance was included in the agenda of Thailand’s eighth national health assembly.^10^ Representatives from academia, civil society and government intensively reviewed the *Draft national strategic plan on antimicrobial resistance* and officially adopted a resolution on the collaborative and comprehensive management of antimicrobial resistance in Thailand. To ensure that the final plan was based on broad stakeholder involvement, the draft plan was discussed by several hundred stakeholders over three public hearings. At the first of these hearings, the members of the antimicrobial resistance coordinating committee introduced the proposed plan’s key features. The plan was drafted in detail at the second hearing and finalized at the third hearing. Antimicrobial resistance is the focus of one of six programmes included in the Thai government’s country cooperation strategy for 2017–2021.

## Relevant changes

After a series of meticulous engagement processes – in particular for identifying potential problems and strategic directions – the Thai government finally endorsed the strategic plan in August 2016 (Box 1). A national steering committee, chaired by the deputy prime minister, is charged with overseeing and coordinating the plan’s implementation and eliminating the fragmentation that hampered previous investigations of antimicrobial resistance in Thailand. It is hoped that the stakeholders’ involvement in the plan’s development will lead to a genuine sense of stakeholder ownership and the plan’s long-term efficacy and sustainability. It is also hoped that the trust-based relationships that exist among the steering committee’s long-serving technocrats who facilitated the plan’s development, i.e. government officials in the ministries of health and agriculture – will strengthen and then also support the plan’s effective long-term implementation. However, it may be difficult to sustain policy commitment because of the typically high turnover of policy-makers.

Box 1 **Actions and targets of the national strategic plan on antimicrobial resistance, Thailand, 2016**

**Actions**

- Strengthen surveillance of resistance, using One Health approach
- Regulate antimicrobial distribution
- Prevent infection in humans while controlling and optimizing use of antimicrobial drugs
- Prevent infection in livestock and pets while controlling and optimizing use of antimicrobial drugs
- Increase public knowledge and awareness of antimicrobial resistance

**Targets to be achieved by 2021**

- 50% reduction in morbidity attributable to antimicrobial resistance
- 20% reduction in mean consumption of antimicrobial drugs by humans
- 30% reduction in mean consumption of antimicrobial drugs by livestock and pets
- 20% increase in the proportion of the population shown to have a pre-defined basic level of knowledge and awareness of antimicrobial resistance
- Capacity of the national plan’s implementation to have reached level 4 – as measured by the World Health Organization’s Joint External Evaluation tool for the 2005 International Health Regulations

The strategic plan is designed to act as a framework that assigns different responsibilities to different institutions and sectors – according to their mandates – with the ultimate aim of achieving set national goals and targets (Box 1).

To strengthen the monitoring and evaluation of antimicrobial resistance and develop the surveillance of antimicrobial consumption, the International Health Policy Program of Thailand’s ministry of public health has convened a series of intensive consultations. These consultations cover multiple stakeholder groups: faculties of pharmacy and veterinary medicine, FAO, several hospitals, Thailand’s department of livestock development, food and drug administration and national laboratory and WHO. The examination of key data sets on antimicrobial sales – i.e. the results of mandatory reporting by importers and manufacturers to the food and drug administration – indicates that it may be feasible to apply similar approaches in Thailand to those used in Europe by the European surveillance of antimicrobial consumption network^11^ and the European surveillance of veterinary antimicrobial consumption project.^12^ An assessment of total antimicrobial consumption by humans, livestock and pets should provide useful data for monitoring the progress of the strategic plan’s implementation.^13^ The magnitude, pattern and types of antibiotic use in pets will be assessed because people commonly use antibiotics created for human consumption to treat their pets. Assessment of the wide use of antibiotics to treat greening diseases in citrus trees is also underway.

Adequate monitoring of the prevalence of antimicrobial resistance in Thailand will require both the existing system for monitoring humans to be strengthened and a whole new system for monitoring livestock and pets to be established. Ideally, given the emergence of resistant foodborne infections in humans, disease surveillance for humans and livestock needs to be integrated.^6^^,^^14^

A new module on antimicrobial resistance, based on a slight adaptation of the format used in the Euro-Barometer 445 survey of such resistance,^15^ has been embedded into Thailand’s biennial national health and welfare surveys, i.e. nationally representative household surveys conducted by the national statistical office. The International Health Policy Program’s long-term institutional relationship with the national statistical office facilitated the inclusion of the new module. The module will be used to assess the prevalence of self-use of antibiotics, knowledge and awareness of antimicrobial resistance and sources of information on antimicrobial resistance. Ideally, all decision-making on antimicrobial use – by clinicians, policy-makers, the general public and veterinarians – needs to be evidence-based.

## Lessons learnt

Strong political commitment, national ownership and increased institutional capacities contributed to the development of the strategic plan (Box 2). Looking ahead, the plan’s effective implementation will require the remaining challenges to be overcome, continued political commitments and a strong technical secretariat. The process of developing the strategic plan placed a high priority on engagement and ownership by key actors, particularly the technocrats who were involved in much of the plan’s contents and who will, probably, be much involved in the plan’s implementation. Given the high turnover of policy-makers, it may be difficult to sustain policy commitment.

Box 2 **Summary of main lessons learnt**

- Strong political commitment, national ownership and adequate multisectoral institutional capacities will be essential for the effective implementation of Thailand’s first national strategic plan on antimicrobial resistance.
- A robust monitoring and evaluation platform now contributes to evidence-based interventions.
- An integrated system for the surveillance of antimicrobial resistance in humans, livestock and pets still needs to be established.
